# ICU and Sepsis: Role of Myeloid and Lymphocyte Immune Cells

**DOI:** 10.1155/2022/7340266

**Published:** 2022-09-25

**Authors:** Anxiu Wang, Su Zhang, Guangming Peng, Yuanxu Tang, Yaopeng Yang

**Affiliations:** ^1^Geriatrics Department, People's Hospital of Yuxi City, Yuxi 653100, China; ^2^Emergency Medicine Department, People's Hospital of Yuxi City, Yuxi 653100, China

## Abstract

Sepsis is a severe immune system reaction to infection and a major cause of ICU-related fatalities. Because of the high mortality, high cost of treatment, and complex aetiology of sepsis, sepsis has a huge impact on healthcare. Some of the health complications in sepsis are abnormal cardiac functions, hypoperfusion, hypotension, tissue damage, multiple organ failure, and ultimately death. Individuals with weak immune systems and chronic medical conditions are highly vulnerable to sepsis. In sepsis, a patient shows the extreme immune response in the initial stage while prolonged immunosuppression in the later stages. Sepsis-driven immunosuppression ushers in death because sepsis cases develop secondary infections postrecovery. The later immunocompromised state in sepsis is attributed myeloid-derived suppressor cell upregulation and reduced immune activity displayed by lymphocytes (lymphocyte anergy). As a result, it is currently suggested that regulating the immune response is a better therapeutic approach than focusing on inflammation to improve the immune system's capacity to fight infections. Moreover, finding novel and accurate prognostic biomarkers that can help in rapid sepsis diagnoses and deciding better therapeutic strategies will significantly lower clinical case mortality rates.

## 1. Introduction

Sepsis reflects a mortality-driving clinical status identified through immunological dysregulation during an infection. Annually, 31,500,000 patients and 5,300,000 mortalities due to sepsis are reported. Septic shock describes a clinical scenario, whereby patient develops vascular-circulatory dysfunction together with profound immune response (IR) against an infection leading to a high mortality. Such IRs are characterized through systemic hyperinflammation early stage, called systemic inflammatory response syndrome (SIRS), together with prolonged immunosuppression during late-phase, termed compensatory anti-inflammatory response syndrome (CARS) [1–4]. Sepsis represents a major mortality driver within severely affected cases residing within intensive care units (ICUs) but degrees of septicemia vary among individuals and depend on the age, overall nutritional status, preexisting medical condition, immune response, and the virulence displayed by the invading pathogen [5]. Sepsis increases the duration of hospital stay, and sepsis patients show 8 times higher mortality than others. According to some estimates, >50% mortality in ICUs are attributed to sepsis [6]. The IR mounted against the infectious agent involves all host immune system components [7]. According to the “host theory” of sepsis, the “cytokine storm,” or unchecked host production of proinflammatory cytokines, is what causes the clinical signs of sepsis [8]. Recent research, however, demonstrates that unchecked production of pro- and anti-inflammatory cytokines is present in this situation. Moreover, cytokine-class also varies among individuals with certain individuals who show increased synthesis of proinflammatory cytokines whereas others show higher production of anti-inflammatory cytokines [8]. The availability of better treatment options has certainly reduced the mortality associated with sepsis, but unfortunately, sepsis survivors are burdened with life-long health complications such as immune dysfunction, increased susceptibility for secondary infections, and poor quality of life [9, 10]. The “postsepsis syndrome” is a relatively new term and indicates a consistent compromised life at cognitive, psychological, physical, and medical level after aggravated sepsis [10]. Several common symptoms of sepsis are tachycardia, tachypnea, body temperature > 38°C or <36°C, WBC-count of >12 × 10^9/^L or <4 × 10^9^/L, and hypotension [11]. Septic condition was found to drastically downregulate circulating numbers of CD4+/CD8+ lymphocytes leading to impaired host IR [7].

The present review is focused on the role of myeloid and lymphocyte cells within immunity issues identified in such cases and several potential biobiomarkers that can be used in improved prognosis and prediction of adverse outcomes during the hospital/ICU admission.

## 2. Etiology of Sepsis

The nonhomeostatic, systemic, and damaging IR that sepsis imposes on the host against infection/s leads to organ failure. Through aberrant stimulation of immune cell components and release of proinflammatory cytokines, the innate immune system supports systemic inflammatory-based responses. The duration and intensity of the inflammatory response have a significant role in sepsis prognosis, and a hyperinflammatory environment is typically associated with negative outcomes [12]. The early IRs in sepsis are mediated by various pattern recognition receptors (PRRs) and pathogen-associated molecular patterns (PAMPs), whereby most share complementary and overlapping functions. PRRs and PAMPs activate host-IR against invading pathogen/s [13]. As per the latest definition of sepsis, the International Consensus for Sepsis and Septic Shock describes it to be a life-threatening condition of organ dysfunction caused by a dysregulated IR to an infection [14]. The diagnosis of sepsis depends upon inflammation-based response-strength within the patient. In sepsis, both overexuberant inflammation and immunosuppression develops simultaneously in a patient. During the initial stages, a sepsis patient generates an inflammatory response against the infection. It progresses towards severe sepsis, a clinical situation in which sepsis is accompanied with organ dysfunction. The final stage is septic shock where a patient develops sepsis with tissue hypoperfusion [15]. The outcome of sepsis cases largely depends on the type of microorganism responsible for sepsis. The European Prevalence of Infection in Intensive Care (EPIC II) investigation reported Gram-negative bacteria are more common in sepsis than Gram-positive species (62.2% vs. 46.8%). The study also noted that the duration of ICU stay increased the risk for sepsis by drug-resistant strains of *Staphylococci*, *Acinetobacter*, *Pseudomonas*, and *Candida* species [16]. Of note, Gram-negative bacteria caused increased mortality in sepsis patients than Gram-positive bacteria [17]. It was observed that *Staphylococcus*/*E. coli* were linked to lower deaths (20% and 19%) than *Candida* (43%) or *Acinetobacter* (40%). The highest mortality (73%) was observed in *Pseudomonas aeruginosa* infections [18]. The lungs are the most frequently colonized site of bacterial colonization, and pulmonary sepsis is more common than abdominal sepsis (56.3% vs. 37.3%), and pulmonary sepsis was more common in old age patients. Both ICU mortality and one-year mortality associated with pulmonary-sepsis prevailed over abdominal sepsis (31.7% vs. 12.6 and 45.4% vs. 24.4%) [19]. One of the major causes for mortality is sepsis which is a multiorgan failure contributed by abnormal activation of blood platelets and immune cells.

An essential mediator in the body's overall response to sepsis is blood platelets. In fact, the main causes of sepsis-induced organ failures are activated platelets and immune system cells. However, a low platelet count is an independent and more potent predictor of poor outcomes in sepsis; therefore, routine platelet testing can aid in accurate risk assessment and the use of alternative therapeutic approaches in the management of sepsis [20]. Sepsis is also more common in individuals with preexisting health complications. For instance, a population level study in the US showed that 16% patients of acute myeloid leukemia (AML) developed sepsis in contrast to 4% patients without AML. The mortality rate of AML patients with sepsis was 30% compared to 21% observed in non-AML patients [21]. Of note, the heightened inflammatory phase in sepsis is followed by an immunosuppression. Recent trends have demonstrated that the immunosuppressive stage of sepsis is the major cause for mortality due to increased risk for secondary infections attributed to “immune paralysis” within a few weeks or month after recovery [22]. This is one of the possible reasons that adjunctive therapy targeted to dampen the inflammatory situation does not yield conclusive results, and the scientific community is of the view that restoration of normal immune functions by utilizing immunostimulants is more promising than anti-inflammatory agents. However, personalized decisions regarding the sepsis therapy must be taken to target inflammation, immunosuppression, or any other metabolism [23].

## 3. Prognostic Biomarkers for Sepsis

A common occurrence in patients with sepsis, trauma, burns, or serious traumas is lymphocyte anergy. Additionally, loss of delayed-type hypersensitivity, which increases the risk of sepsis and death [24], is linked to lymphocyte anergy. The neutrophil-to-lymphocyte ratio (NLR) is an important indicator for sepsis prognosis. A significantly higher ratio of NLR is observed in sepsis nonsurvivors together with exacerbated NLR linked to poor prognoses in septic cases [25]. Additionally, a higher NLR ratio was observed within acute kidney injury (AKI) patients in sepsis and acted as an independent indicator for AKI within sepsis/septic-shock cases [26]. Statistically validated variations within salivary C-reactive protein levels within septic neonates were denoted, in comparison to control cohort (12.0 ± 4.6 ng/L versus 2.8 ± 1.2 ng/L). Moreover, the salivary CRP levels were also a good indicator of subsequent rise in the serum CRP levels within such cases. Furthermore, the mean platelet volume and NLR were also markedly exacerbated within such cases in comparison to control cohort [27]. Recently, neutrophils-to-lymphocytes-and-platelets (N/LP) ratio was also suggested as proxy prognostic biomarker for inflammatory stati in sepsis. In patients with AKI, an exacerbated ratio of N/LP indicated aggravated danger of death and a separate prediction biomarker for death within septic-AKI cases admitted to ICUs [28]. The soluble triggering receptor expressed on myeloid cells-1 (sTREM-1) represent valuable biomarkers for understanding sepsis/septic shock intensities. Additionally, it helps distinguish between septic and nonseptic illnesses. Compared to CRP and procalcitonin, sTREM-1 is thought to have improved sensitivity and specificity, making it a viable biomarker for the quick identification of infectious illnesses [29]. Saldir et al. reported septic neonates showed markedly exacerbated levels of IL-6, sTREM-1, endocan, and immature/total neutrophil ratio (I/T ratio) than nonseptic neonates. The measurement of these biomarkers can help in early identification of sepsis in neonates. The study showed that IL-6 was the most accurate biomarker for sepsis followed by sTREM-1 [30]. Plasma levels of sTREM-1 were markedly exacerbated within sepsis, compared to SIRS. Moreover, plasma sTREM-1 levels varied within (severe) sepsis/septic shock cases. This indicates that sTREM-1 is a functional biomarker for sepsis progression and a direct indicator of disease severity [31]. Another useful biomarker for sepsis is myeloid-related protein complex 8/14 because MRP8/14 expression levels increase with sepsis severity. The nonsurvivors had an exacerbated level of MRP8/14 than survivors in a 28 day follow-up. Moreover, AKI-carrying sepsis cases showed upregulated MRP8/14 than patients without AKI. This indicated that MRP8/14 acts as a functional biomarker for sepsis diagnoses/progression in ICU cases exhibiting AKI [32]. Recent observations have shown that the ratio of platelet-to-lymphocyte (PLR) is an important prognosis biomarker regarding inflammation in sepsis. A PLR > 200 indicated markedly exacerbated mortality, and a ratio of PLRs ≤ 200 was not significant [33].

## 4. Myeloid Cells in Sepsis

Myeloid-derived suppressor cells represent Gr1^+^ CD11b^+^ immune components defined through reduced expression of several characteristic biomarkers used to classify mature myeloid cells. They are also known as null cells, myeloid suppressor cells, or immature myeloid cells. These elements are the granulocyte and monocyte progenitors, and they may suppress the T cell response during an inflammatory state. To avoid any confusion, such immunological components were given the generic designation myeloid-derived suppressor cells (MDSCs) in 2007 [34, 35]. MDSCs express Gr1 and CD11b, two myeloid differentiation biomarkers, and first identified their crucial role in antitumor and immune surveillance. The heterogeneous population of MDSCs comprises the precursor of immune components including dendritic cells, macrophages, together with granulocytes, strongly inhibiting T cell function by exacerbating nitric oxide and reactive oxygen species generation [36]. Apart from their central role in immunosuppression, MDSCs also play certain nonimmunological roles in tumor angiogenesis and tumor metastasis [37, 38]. MDSCs are immunosuppressive, and their number increases in medical conditions characterized by acute or chronic inflammatory milieu such as cancer. Recent studies have linked MDSCs to the pathogenesis of sepsis. Strikingly, an increased number of MDSCs were responsible for nosocomial infections, adverse outcomes in sepsis patients, and exacerbated mortality in ICU admitted sepsis patients. Since MDSCs are present in very low numbers in healthy subjects, such could be employed as biomarkers and drug-action sites in sepsis therapy [39]. Primary function of MDSCs is immunosuppression by controlling the inflammation in sepsis. The role that this MDSC function plays in sepsis is dual. The host immune system mounts a potent IR during the early stages of septic shock, which causes hyperinflammation. Immunosuppression brought on by MDSCs during this phase prevents organ malfunction and restricts the harmful effects of hyperinflammation. In contrast, persistent inflammation-immunosuppression and catabolism syndrome (PICS) and chronic critical illness (CCC) are both brought on by long-term immunosuppression brought on by MDSCs [40]. During the first three days of the septic phase, MDSCs were produced. These cells enhanced proinflammatory cytokine populations, released nitric oxide, and raised mortality. However, MDSCs in the late phase of sepsis (12 day) are anti-inflammation, expressing IL-10/TGF-beta. Late MDSCs showed more immature phenotype than early MDSCs and created less macrophages/dendritic cells in comparison to primordial MDSCs when treated with GM-CSF. This suggests that as septic inflammation developed, MDSCs skew towards a more immature phenotype and change their nature from proinflammatory to anti-inflammatory immune cells [35]. MDSCs suppress the activity of both adaptive and innate immune systems and promote chronic immunosuppression observed across late septic phases [41]. MDSCs were upregulated during several health complications where acute or chronic inflammatory conditions are a common underlying cause. For instance, sepsis, autoimmune disorders, burns, cancer, and trauma are certain clinical conditions where MDSC numbers increase. MDSCs are powerful immunosuppressive immune-system components stemming from their ability to reduce the suppress CD8(+) and CD4(+) T cell activation [42]. MDSC-induced immunosuppression is attributed to degradation of L-arginine, discharging anti-inflammatory/immunosuppressive cytokines such as IL-10 and TGF-*β*, activating immunosuppressive T regulatory cells (T_regs_) and exacerbated generation of reactive oxygen and reactive nitrogen species (ROS, RNS) [39]. Xu et al. reported that 95% of esophageal tumor cases displayed upregulated granulocyte derived-MDSCs (G-MDSCs), correlating with elevated postsurgical morbidities. Moreover, an exacerbated number of monocyte-derived MDSCs indicated poor prognosis in cancer-related sepsis [43]. At molecular level, generation of MDSCs is linked with miR-21 and miR-181b expressions. The CCAAT enhancer-binding protein (C/EBP*β*) upregulated miR-21 and miR-181b, leading onto transcription factor NFI-A upregulation and promoting MDSCs within spleen/bone marrow within a murine model for sepsis. However, C/EBP*β*-deficient myeloid progenitors showed reduced NFI-A and consequently reduced generation of MDSCs in septic mice. This suggests that reducing the expression of C/EBP*β* can be used as a therapeutic strategy to reduce immunosuppression in sepsis treatment [44]. In mouse model of Gram^+^ sepsis, a massive upregulation in Gr1^+^ CD11b^+^ MDSC populations was observed. Both G-MDSCs (Ly6G^−^ CD11b^+^) and M-MDSCs (Ly6C^+^Ly6G^−^CD11b^+^) were increased but M-MDSCs showed a stronger increase in the numbers for longer duration than G-MDSCs. At molecular level, the postseptic immunosuppression is mediated by IL-6-dependent MyD88 and TLR signaling [45].

## 5. Lymphocytes in Sepsis

The adaptive immune system's B and T lymphocytes are crucial parts because they trigger an antigen-specific immune response to an invading disease. B (humoral immunity) and T cells are required for the initial antigen recognition and subsequent IR to eliminate the foreign antigen (cell-based immunity). While B cells differentiate into plasma cells and generate bespoke antibodies to clear infections, T cells are responsible for cell-mediated clearance of the invading pathogen [46]. Sepsis is characterized by reduced numbers of both B and T lymphocytes, a clinical condition called B and T lymphopenia, which causes immunosuppression in the patient. The B and T lymphopenia in sepsis is attributed to extensive apoptosis of lymphocytes, and preventing lymphocyte apoptosis by using caspase inhibitors markedly reduced the mortality in sepsis [7]. Anergy is a tolerance mechanism in immune cells where the cells do not mount a normal IR against an antigen. The cells remain in an inactivated but live stage for a prolonged duration in a hyporesponsive state [47]. Anergy of T lymphocytes is associated with immunodepression and indicates loss of activation through TCR signaling or Ca^(2+)^ mobilization [48]. CD4^+^, CD8^+^, and total T lymphocyte downregulations were reported within sepsis cases. Lymphocyte downregulation was, however, induced by the type of bacterial infection, and Gram-negative bacteria more severely suppress the immune system than Gram-positive bacteria. For instance, sepsis induced by Gram-positive bacteria *Streptococcus pneumoniae* and *Staphylococcus aureus* caused an extended reduction (≥14 days) for CD4^+^, CD8^+^, total T lymphocyte, and NK cellular populations. Conversely, sepsis by Gram-negative pathogens, *Neisseria meningitidis* and *Enterobacteria*, caused reduction for a smaller duration, and the patients fully recovered in 3 days. Moreover, B cell/CD3^+^/DR^+^ and CD4^+^ T lymphocyte populations within *Neisseria meningitidis* and *Enterobacteria*-infected patients were rapidly and markedly increased during the recovery phase compared to Gram-positive septic cases [49]. Population statistics for total T lymphocytes and CD4+ T lymphocytes were markedly reduced within septic patients than normal controls. Moreover, septic patients also showed lower numbers of NK cells, CD3+/DR + lymphocytes and CD4/CD8 ratio than healthy controls. However, the number of B lymphocytes was increased [50]. Treatment with Rg1 markedly increased the survival rate by suppressing systemic inflammatory response and enhancing the bacterial clearance. Moreover, Rg1 also inhibited lymphocyte apoptosis and attenuated lung and liver injury in septic mice which suggests that Ginsenoside Rg1 is protective in CLP-induced polymicrobial sepsis due to its anti-inflammatory and immunomodulatory activities [51]. Sepsis-induced lymphopenia was observed in patients during a 28-day follow-up. Sepsis nonsurvivors lowered degrees of CD19+ CD23+ across a one-week follow-up compared to sepsis survivors and a CD19+CD23+ value of 64.6% on receiver-operating characteristic curve was able to discriminate between sepsis nonsurvivors and sepsis survivors. Moreover, sepsis nonsurvivors showed an exacerbated percentage of CD80+ and CD95+ B cells than survivors. This suggests that a lower percentage of CD23+ and exacerbated CD80+ and CD95+ B cell percentages were linked to exacerbated death incidences during ICU admission in septic shock patients [52].

Recently, a “lymphocyte apoptosis model” is proposed to stratify risks together with improving prognoses within septic cases. The model is based on the biomarkers for lymphocyte apoptosis/immune-function and have potential in predicting survival in septic cases. The study observed that on the day 1 of admission, sepsis perishers showed markedly exacerbated levels of lymphocyte apoptosis and plasma cytochrome C, together with markedly reduced lymphocytes, Th1/Th2 ratios, and HLA-DR expression than sepsis survivors [53].

It has been observed that ICU-admitted septic cases showed reduction in all major lymphocytes: B, T, and NK cells. Moreover, critically ill patients also showed downregulated T cells together with a markedly reduced ICU mortality was observed in patients which showed an exacerbated total T cell count (>0.36/nL) on ICU presentation, independent of the patient's age. Also, sepsis survivors showed restoration of lymphocytes, and T cells and sepsis perishers were failed to overcome lymphopenia and T cell depletion [54].

## 6. Therapeutic Approach to Combat Sepsis

For many years, an uncontrolled inflammation was considered as the major cause for sepsis-associated symptoms including pyrexia and respiratory distress, together with shock. Further supporting our belief that targeting inflammatory pathways to minimise cytokine storm is the key to combating sepsis and lowering sepsis-related mortality is the finding that proinflammation cytokines like TNF- and IL-1 become elevated within sepsis. Alternative therapeutic approaches are required to treat septic cases, as shown by clinical trials targeting inflammatory pathways that either failed or even reduced the survival rate in septic patients. In actuality, immunoparalysis—which is a direct result of elevated lymphocyte apoptosis—causes the majority of sepsis individuals to pass away [55]. This suggests that preventing immunosuppression by reducing lymphocyte apoptosis is a promising strategy to reduce late phase complications in sepsis. Several studies have been undertaken to study the therapeutic potential of natural products and supplements. For instance, genipin treatment reduced late-phase lymphocyte apoptosis by reducing the expression of FADD, caspase-8, and caspase-3 and consequently increased the survival rate of mice in the CLP model of sepsis. Moreover, genipin prevented a reduction in the numbers of CD4^+^ and CD8^+^ T cell population and reduced the number of immunosuppressive regulatory T cell (Treg). Genipin also reduced immunosuppression by increasing the splenic expression of interferon-*γ* and interleukin (IL)-2 and reducing the levels of IL-4 and IL-10 [56]. In a related study, ASI-IV therapy enhanced overall survival in septic mice, decreased pathological damage to the lung and spleen, suppressed NF-B and ERK1/2 signalling pathways, decreased bacterial load, and decreased levels of proinflammatory cytokines. These biological effects of ASI-IV protected mice against sepsis [3]. Glutamine also protected against sepsis-induced inflammatory reactions by increasing the numbers of CD8*αα*^+^ TCR*αβ*^+^ IELs and reducing the apoptosis in these cells. Glutamine also reduced the expression of proinflammatory cytokines from CD8*αα*^+^ TCR*αβ*^+^ IELs and mitigated intestinal epithelial injury during sepsis [57]. Martire-Greco et al. reported reduced lymphocytes and increased MDSCs in the lymph nodes of immunocompromised mice along with abnormal T cell proliferation. However, treatment with all-trans-retinoic acid (ATRA) restored the immunocompetence by increasing the numbers of CD4^+^ and CD8^+^ T cells which consequently improved the humoral immunity indicating that ATRA administration can be a promising strategy to ameliorate the immunosuppressive state in septic cases [58].

## 7. Conclusion

An early inflammatory condition and a subsequent yet lingering immunosuppressive condition define sepsis, an inflammatory clinical condition. The biochemical pathways and immunological cells involved in the genesis of sepsis make up a very complex network. Because of its complicated origin, high death rate, and dearth of effective treatments, sepsis places a heavy burden on healthcare systems. The advanced stages of sepsis can cause organ dysfunction and ultimately death. In sepsis, a cytokine storm is followed by a compromise immunity called immunoparalysis which increases the chances of secondary infection and associated mortality. Sepsis remains a main driver for mortalities within ICU-admitted cases, and early diagnosis and appropriate treatment options can improve prognosis and reduced mortality. Moreover, early prognosis also helps in choosing appropriate antibiotics which can improve treatment outcomes. In recent years, it has been established that a majority of septic patients succumb to their illness due to immunocompromised state and not due to hyperinflammation. MDSCs are a mixed combination of myeloid cells and reduce the IR by inhibiting T cell-based immunity. The severely immunocompromised state in sepsis is attributed to increased numbers of MDSCs and lower levels of various types of lymphocytes. In contrast, some studies have even suggested that absence of MDSCs remains a main driver for mortalities within sepsis. This suggests that the role and immune functions of MDSCs want further exploration due to their controversial role in sepsis. Although treating infections and other sepsis-related complications continues to be the mainstay of treating sepsis, recent developments in the study of immune cells, such as MDSCs and lymphocytes, have opened the door for newer therapeutic approaches to combat the condition's immunosuppressive state. Numerous studies have demonstrated that one method for overcoming reduced IR in septic infections is to reduce lymphocyte apoptosis. Additionally, the strategies are aimed at reducing secondary infections in septic cases postrecovery which must also be explored for lowering mortality and enhancing quality-of-life in septic cases postrecovery.

## Data Availability

Data will be provided upon request.
